# First Do No Harm: Legal Principles Regulating the Future of Artificial Intelligence in Health Care in South Africa

**DOI:** 10.17159/1727-3781/2022/v25ia11118

**Published:** 2022-04-07

**Authors:** Dusty-Lee Donnelly

**Affiliations:** School of Law, University of Kwa-Zulu Natal, South Africa.

**Keywords:** Artificial intelligence, ethics, health care, health policies, machine learning

## Abstract

What sets AI systems and AI-powered medical robots apart from all other forms of advanced medical technology is their ability to operate at least to some degree autonomously from the human health care practitioner and to use machine-learning to generate new, often unforeseen, analysis and predictions. This poses challenges under the current framework of laws, regulations, and ethical guidelines applicable to health care in South Africa. The article outlines these challenges and sets out guiding principles for a normative framework to regulate the use of AI in health care. The article examines three key areas for legal reform in relation to AI in health care. First, it proposes that the regulatory framework for the oversight of software as a medical device needs to be updated to develop frameworks for adequately regulating the use of such new technologies. Secondly, it argues that the present HPCSA guidelines for health care practitioners in South Africa adopt an unduly restrictive approach centred in the outmoded semantics of telemedicine. This may discourage technological innovation that could improve access to health care for all, and as such the guidelines are inconsistent with the national digital health strategy. Thirdly, it examines the common law principles of fault-based liability for medical negligence, which could prove inadequate to provide patients and users of new technologies with redress for harm where fault cannot clearly be attributed to the healthcare practitioner. It argues that consideration should be given to developing a statutory scheme for strict liability, together with mandatory insurance, and appropriate reform of product liability pertaining to technology developers and manufacturers. These legal reforms should not be undertaken without also developing a coherent, human-rights centred policy framework for the ethical use of AI, robotics, and related technologies in health care in South Africa.

## 1 Introduction

From time immemorial doctors have sworn to treat their patients to their greatest ability and to do them no harm. This spirit is retained in the revised Geneva declaration in which doctors also pledge to respect patient autonomy and dignity, eschew discrimination, and maintain patient confidentiality while sharing their medical knowledge in the interests of the patient and the advancement of medicine.^1^ But how do regulators ensure that autonomous artificial intelligence (AI) systems, medical robots and related technologies are designed to obey the same laws and ethical codes? This is an urgent question as AI is set to play a growing role in all aspects of public and private health care and health research, including the making of great advancements in clinical diagnostics and decision-making and health care management. For example, during the COVID-19 pandemic AI facilitated disease surveillance and outbreak monitoring across the globe.

The capacity of AI systems to operate at least to some degree autonomously from the human health care practitioner and to use machine-learning to generate new, often unforeseen analyses and predictions is what sets AI systems and AI-powered medical robots apart from all other forms of advanced medical technology. A key priority is to develop laws and policy to support the “ethical and transparent use” of these new technologies,^2^ and the transparent and secure management of health data sets on which algorithmic models can be built.^3^

While a core set of general principles for the ethical development of AI has emerged,^4^ those principles must still be operationalised through legal regulations,^5^ and this is particularly important in a high-risk area such as health care. The enactment of comprehensive data protection laws, while important, is not sufficient to address the unique regulatory challenges posed by AI.^6^ South Africa has no laws specifically regulating AI.^7^ Thus existing legal principles must be adapted, or new principles developed to mitigate the risks to human well-being (comprising of both health-related and human rights-related risks) while not stifling innovation and leading (unintentionally) to non-compliance.^8^

This article examines the extent to which current South African laws and policy in health care align with the normative framework of international principles for ethical AI and the values underpinning South Africa’s constitution. It examines three legal issues central to the effective regulation of AI: the regulatory oversight mechanisms for the registration of new AI health technologies, the health professions ethics framework governing the use by health care practitioners of these new technologies, and the common law principles of liability for harm caused to a patient or user of the technology. It concludes with recommendations for the development of a clear AI strategy with clear ethical guidelines centred in a human-rights narrative for the implementation of AI in health care in South Africa.

## 2 Artificial intelligence: the future for health care in South Africa

Artificial intelligence is expected to boom in Africa in the coming years.^9^ AI could help to address a lack of access to health care facilities and a shortage of skilled health care practitioners, and lead to advances in health care policy and delivery through the better prediction, prevention, diagnosis and treatment of disease.^10^ But despite these possibilities AI is “rarely deployed in medical practice, due to technical, regulatory and ethics concerns”,^11^ and in Africa it is also being held back by a lack of access to the robust open data sets on which the development of AI depends.^12^

The primary application of AI in health care considered in this article concerns patient interactions that are directly mediated by a human health care practitioner who is assisted by AI. For example a KwaZulu-Natal Department of Health initiative to meet UNAIDS’s^13^ “90-90-90 target” in the treatment of HIV/AIDs empowers rural health care workers and Department of Health Services administrators with AI-powered insights through Vantage, a South African information and communications technology (ICT) start-up.^14^ The project is just one example of the potential of AI to increase the ability of health care practitioners to mediate successful patient outcomes, and the synergy between the policy goals of improving the conditions of each South African, and empowering small, medium and micro-sized enterprises (SMMEs) to work competitively in the ICT sector, not simply as consumers of technology but as developers of innovative new applications of technology.^15^ AI has innumerable promising applications in health care, ranging from the interpretation of medical images, laboratory results and time series data, to biomedical text mining, electronic health record analysis and medical decision support systems.^16^

## 3 Defining key terms for a new regulatory framework

Artificial intelligence has not yet been authoritatively defined. The European Union (EU), which is currently at the most advanced stage worldwide in the development of AI laws and regulation,^17^ has proposed that it be defined as:

a system that is either software-based or embedded in hardware devices, and that displays intelligent behaviour by, inter alia, collecting, processing, analysing, and interpreting its environment, and by taking action, with some degree of autonomy, to achieve specific goals.^18^

The Organisation for Economic Co-operation and Development (OECD) has adopted a similar definition:

An AI system is a machine-based system that can, for a given set of human-defined objectives, make predictions, recommendations, or decisions influencing real or virtual environments. AI systems are designed to operate with varying levels of autonomy.^19^

AI now uses big data analytics^20^ powered by complex algorithms^21^ to collect and interpret data. The term “algorithm” refers to the computational process or set of coded “instructions” that will be implemented by the computer programme to perform a function or solve a problem.^22^ However, new machine-learning (ML) techniques^23^ enable AI to “complete tasks in a way that would be considered intelligent were they to be completed by a human”^24^ as the machine can move beyond a coded set of instructions to adapt and improve as it “learns” from the data.^25^ In a health care setting one can distinguish broadly between ML techniques applied to the analysis of structured data, such as imaging, genetic and electrophysiological data, and natural language processing techniques used to analyse unstructured data, such as clinical notes in digitised health records, and generate machine-readable structured data for further analysis.^26^ In both instances the “deep learning” enabled by adaptive algorithms means that the manner in which the machine responds to data is no longer pre-determined and entirely predictable.^27^

Similarly, advances in ML mean that one must now distinguish between “deterministic” robots, which can act autonomously but will do so in a predictable manner determined by pre-programmed instructions, and “cognitive” robots, which are powered by stochastic or adaptive algorithms that enable the robot to take decisions based on the input it receives from its environment but means that the robot’s actions are not always predictable.^28^

## 4 Normative framework for ethical AI development

The development of specific laws to regulate AI remains in its infancy. Although the Council of Europe’s *ad hoc* committee on AI (CAHAI) has put forward a proposal for an AI treaty, the work planned for 2021 remains at the stage of a study of its feasibility and scope.^29^ However, guiding normative principles have been developed by several international organisations and are largely convergent, emphasising respect for human rights and freedoms^30^ alongside transparency, fairness, security and, more broadly, beneficence and accountability as core components of ethical AI development.^31^ These values are encapsulated in the OECD’s five Principles on AI:^32^

- AI should benefit people and the planet by driving inclusive growth, sustainable development and well-being.
- AI systems should be designed in a way that respects the rule of law, human rights, democratic values and diversity, and they should include appropriate safeguards – for example, enabling human intervention where necessary – to ensure a fair and just society.
- There should be transparency and responsible disclosure around AI systems to ensure that people understand AI-based outcomes and can challenge them.
- AI systems must function in a robust, secure and safe way throughout their life cycles and potential risks should be continually assessed and managed.
- Organisations and individuals developing, deploying or operating AI systems should be held accountable for their proper functioning in line with the above principles.

As a member of the United Nations Educational, Scientific and Cultural Organisation (UNESCO), it is to be expected that South Africa will be guided in its national legislative and policy development agenda by the Recommendation on the Ethics of Artificial Intelligence adopted by UNESCO’s General Conference at its 41st session on 24 November 2021.^33^ In addition, as a member of the G20 South Africa should take guidance from the G20 AI principles^34^ adopted in 2019, which are in turn modelled on the OECD Principles on AI. These principles strongly overlap with the EU framework for “trustworthy AI”,^35^ the United Nations Educational, Scientific and Cultural Organization (UNESCO) recommendation,^36^ and industry-led commitments to ethics such as those of the IEEE,^37^ Microsoft,^38^ Google^39^ and DeepMind.^40^

However, differences in how these “soft” principles are interpreted and the extent to which they are applied by corporate actors^41^ require the development of enforceable obligations in laws, regulatory policy and professional codes of conduct.

## 5 South African legislative and regulatory policy framework for AI in health care

The artificial intelligence applications developed for or used in a health care setting must operate in full compliance with the *National Health Act* 61 of 2003, the *Health Professions Act* 56 of 1974, the *Medicines and Related Substances Act* 101 of 1965 and the *Hazardous Substances Act* 15 of 1973. In addition, legislation governing consumer products or services,^42^ the protection of personal information,^43^ access to personal information^44^ and electronic transactions^45^ must be applied where relevant. The development of policies, standards, and certification mechanisms for AI applications in health care will thus require constructive dialogue and co-ordinated action by the Information Regulator, the Department of Health (DOH), the South African Health Products Regulatory Authority (SAHPRA) and other stakeholders in South Africa’s digital health strategy.^46^

### 5.1 Artificial intelligence in digital health policy

South Africa adopted a telemedicine strategy in 1998 but failed to achieve the targeted improvements in access to health care in under-resourced rural communities that telemedicine promised.^47^ Policymakers have since set their sights even higher on a global digital health strategy led by the World Health Organisation (WHO),^48^ which still includes telemedicine in the broader rubric of e-health,^49^ but now also includes 4IR technologies such as AI, big data analytics and robotics.^50^ At a regional level digital health is also a key pillar in the African Union (AU)’s Digital Transformation Strategy,^51^ and the Policy and Regulation Initiative for Digital Africa (PRIDA) is developing Africa’s digital health strategy.^52^

South Africa’s latest digital health policy strategy adopts the WHO definition of digital health^53^ and therefore sets a clear green light for the development and deployment of AI applications in health care in pursuit of the strategic vision and detailed objectives of the policy. But the policy itself and the existing legislative and regulatory policy environment in South Africa are lacking in substantive principles to guide such development or deployment.

The term “health technology” refers to “machinery or equipment that is used in the provision of health services”,^54^ excluding medicines.^55^ At national and provincial level, the Health Council is to advise the Minister of Health on

policy concerning any matter that will protect, promote, improve and maintain the health of the population, including- … (v) development, procurement and use of health technology.^56^

The acquisition of any “prescribed health technology” by a health establishment is subject to the issue of a certificate of need by the Director-General.^57^ The Minister of Health, after consultation with the National Health Council, may promulgate regulations^58^ and prescribe quality requirements and standards relating to health technology,^59^ and the Office of Standards Compliance and the Inspectorate for Health Establishments must monitor and enforce compliance by health establishments with such standards.^60^ The framework thus exists in which the use of AI in health care could be evaluated, but it continues to face challenges in implementation.^61^

### 5.2 Artificial intelligence software as a medical device

The *Medicines and Related Substances Act* 101 of 1965, as amended,^62^ defines the term “medical device” widely to include *inter alia* any “machine” and “software” intended by the manufacturer for use in the “diagnosis, treatment, monitoring or alleviating” of any disease or injury, and the “prevention” of any disease. Many but not all possible applications of AI in the field of health care will fall within this definition,^63^ including software that can assist with diagnosis in a clinical setting, and the hardware embedded with AI software that makes robotic surgery assistants, nursing aides and nano-robots possible. In both examples the AI software is clearly intended by the manufacturer to be used for the medical purposes defined. General software that is not specifically intended for such a purpose is not a medical device, “even if it is used in a health care setting.”^64^

The lines become blurred in the area of smart wearable devices and “fitness” and “health” mobile apps for smartphones, which may be considered “lifestyle” or “general wellness” products that mostly fall outside the ambit of health care regulations.^65^ So, too, a chatbot developed in Kenya to offer sexual and reproductive health care information (but not medical “advice”) and the chatbots developed during the COVID-19 pandemic to provide symptom checking, reporting and exposure services would not *prima facie* be classified as medical devices as they are not being used in the diagnosis of disease (or a prescribed course of treatment). Nevertheless, there can be clear health implications if these chatbots incorrectly direct a patient, raising ethical concerns and the question of how they should be regulated to prevent the risk of harm.^66^

However, the involvement of a human health care practitioner is not a requirement imposed by the definition of software as a medical device under the *Medicines and Related Substances Act* 101 of 1965. Thus, currently medical devices intended for self-monitoring by a patient, for example blood pressure monitors or blood glucose tests, fall within the definition. It is conceivable that in future AI-powered devices that provide an interpretative analysis of data for a diagnosis of the underlying disease or injury would fall within the definition, provided the device is objectively intended by the manufacturer to be used in this way.

Interpretative clarity on the ambit of the definition is essential to ensure that the developers of such software are directed to appropriately consider the risks posed by the software and to implement a quality management system for the software lifecycle, which is especially important when software is used outside of a clinical setting.

### 5.3 The need for reform of regulatory oversight mechanisms

Medical devices that meet defined “standards of quality, safety, efficacy and performance”^67^ are registered by SAHPRA after evaluation and assessment. SAHPRA may declare that a medical device (or any class, or part of any class, thereof) must be registered.^68^ The sale of any medical device that has not been registered as required by such a declaration is prohibited.^69^ The process by which applications for registration are reviewed by SAHPRA is governed by section 15 of the *Medicines and Related Substances Act* 101 of 1965, and requires SAHPRA to receive particulars and “where practicable” samples of the medical device.

This single stage model for regulatory review according to pre-defined, static specifications and standards cannot adequately address safety, quality and efficacy concerns as AI systems are “adaptive”, with the software algorithms being trained from large data sets so that the machine may change its behaviour over time in response to new insights learned from real-world applications.

The United States Food and Drug Administration (FDA) have proposed a “total product lifecycle”^70^ regulatory oversight mechanism for software such as medical devices in health care. Pre-market certification of software would require manufacturers to provide the FDA with a “pre-determined change control plan” outlining the modifications that can be anticipated, coupled with transparent monitoring throughout the product lifecycle.^71^

In the EU, Regulation 2017/745 on medical devices^72^ expands the definition of medical device to include the “prediction and prognosis” of disease, which may bring certain mobile applications such as heart rate monitors on smartphones and smartwatches into the regulatory regime.^73^ Further, a specific classification standard for software has been introduced.^74^ To complement sectoral product safety legislation the EU has also adopted a proposal for an AI Act to regulate the conditions applicable to the development and marketing of all AI-products and services and has established post-market controls.^75^

At an international level the Focus Group on AI for health (FG-AI4H), established in 2018 by the International Telecommunications Union (ITU) in partnership with the World Health Organization (WHO), provides

a standardized assessment framework for the evaluation of AI-based methods for health, diagnosis, triage or treatment decisions.^76^

In 2021 the WHO published a framework to guide the evaluation of clinical evidence supporting AI software development, software validation and reporting, deployment, and post-market surveillance.^77^ The framework is a ground-breaking development that will assist in ensuring that safety and performance claims are supported by robust, transparent evidence. Importantly it emphasises that evidence must be free of the existing biases in healthcare on racial, ethnicity, age, socio-economic and gender lines that are perpetuated when they are encoded into the data used to train AI algorithms.^78^

It is essential that consideration be given to these developments to reform the regulatory regime in South Africa.^79^ Public authorities must have oversight and the ability to intervene at all stages of the AI product lifecycle. The development of technical standards, robust ethical guidelines and a certification process could be considered as means to ensure oversight before market launch, so that health care practitioners and patients have access to trustworthy AI products and services only.

In the case of high-risk use, where indicated by a risk assessment, there would be a general obligation upon developers to deposit the documentation on the use, design and safety instructions with public authorities, and where “strictly necessary” this might include information on the “source code, development tools, and data used by the system”.^80^ Allowing authorities access to the data, software and computer systems of developers and deployers of AI technologies is necessary to verifying not only the intended purpose but also the actual uses to which AI is put.^81^ Such access must of course take place with safeguards to protect data, privacy, intellectual property rights and trade secrets.^82^ In this regard, without duplicating duties, there needs to be co-operation between the Information Regulator and the health sector regulatory bodies to ensure that new technologies identified as “high risk” are developed and deployed in accordance with legal and ethical obligations^83^ and an approved certification process.^84^ Consideration also needs to be given to support for end-of-life products, and “independent trusted authorities” must have the means to provide services such as maintenance, repair and software updates and patches to the users of “vital and advanced medical appliances” where the developer or deployer of the technology ceases to do so.^85^

### 5.4 Need for regulatory reform of ethical guidelines

The Health Practitioners Council of South Africa (HPCSA)’s ethical guidelines for practitioners remain rooted in the outdated era of telemedicine.^86^ Telemedicine is defined in the guidelines as:

The practice of medicine using electronic communications, information technology or other electronic means between a health care practitioner in one location and a health care practitioner in another location for the purpose of facilitating, improving and enhancing clinical, educational and scientific health care and research, particularly to the under serviced areas in the Republic of South Africa.^87^

Thus, telemedicine seeks to replicate traditional face-to-face practitioner-patient consultations using ICTs such as video conferencing. It could also include the exchange of information electronically (between practitioner and patient or, for example, between the primary and secondary health care practitioner for a specialist diagnosis or a second opinion) but an

actual face-to-face consultation and physical examination of the patient in a clinical setting by at least one of the health care practitioners remains mandatory.^88^

The guidelines are further restricted by the requirement that both the consulting practitioner and the servicing practitioner must be registered health care practitioners, either in South Africa or in the country where they are located.^89^ A medical examination must be performed and documented, with a clinical history of the patient, before any course of treatment is prescribed or prescription issued.^90^ No course of treatment or prescription may be issued on the basis of a questionnaire alone,^91^ and informed consent must still be obtained when a prescription is issued electronically.^92^ The guidelines have been relaxed recently, but only for the duration of the COVID-19 pandemic, and only to the extent of permitting “telehealth”^93^ even where there is not “an already established practitioner-patient relationship”.^94^

The HPCSA ethical guidelines are thus inadequate to regulate the lawful and ethical development and deployment of AI applications. Worse, they may in fact inhibit the adoption of new technologies in health care in South Africa by virtue of the threat of sanctions against health care practitioners if they are found guilty of unprofessional conduct^95^ or a breach of the professional duties imposed by common law.^96^ The HPCSA’s statutory mandate under section 3 of the *Health Professions Act* 56 of 1974 is subordinate to national health laws and policy. Presently the outdated guidelines are inconsistent with the national policy on digital health, which includes innovation through the adoption of new technologies such as AI as one of five key principles underpinning the strategy.^97^ While the report of the Presidential Commission on the Fourth Industrial Revolution (4IR) recognises that there remains a role for telemedicine in bridging disparities in physical access to health care services,^98^ it underscores the need to leverage new technologies such as AI for efficiency and cost saving in health care planning, as well as advancements in the medical treatment of patients.^99^

Although machine-learning has transformed the role of the medical device from a mere tool to a powerful collaborator with the health care practitioner,^100^ there is no room in the guidelines to regard an AI system as a servicing practitioner working in partnership with the consulting practitioner.^101^ While South African law recognises juristic persons, it does not presently afford any legal status to “things”.^102^ A radical re-imagining may be necessary to address the new risks and roles of AI and there is, at least in principle, no reason why a statute cannot create a statutory right of action against an AI system (the thing) which would impeach it (without necessarily citing or requiring jurisdictional competence over the person who owns or operates the thing).^103^ However, without comprehensive, insurance-backed provisions for recourse in the event of harm, such provisions may be meaningless.

## 6 Guiding principles for the development of civil liability for medical harm in an AI context

As a corollary to the development of a regulatory oversight and professional ethics framework for the development and use of AI, consideration must be given to the basis upon which civil liability may be attributed when technology fails and causes harm. In this section two guiding principles are put forward to guide future regulation in this area.

### 6.1 Informed consent from the patient must always be obtained

Informed consent is the bedrock to the provision of any health care service. Sections 6 and 7 of the *National Health Act* 61 of 2003 respectively provide the way a patient is to be informed, and stipulate that a health service may not be provided to a user without that user’s informed consent, save in limited exceptional circumstances.^104^ In terms of section 7(2),

[a] health care provider must take all reasonable steps to obtain the user’s informed consent.

The only guidance available on the use of technology in a health care setting is that in addition to obtaining the patient’s informed consent to a prescription or any course of treatment, the patient must also give informed consent to the use of the technology.^105^ While the technologies underlying telemedicine such as video conferencing and email are now so commonplace that one can see little difficulty in providing an understandable explanation to the patient, the same cannot be said about AI. While this may change somewhat as new technologies infiltrate all areas of daily life, it is unlikely to ever be the case that an average patient will understand the complex algorithms that power AI systems. The scholarly debates taking place around the legal requirement for “transparency”^106^ (or “explainability”)^107^ must be tempered by pragmatism. Just as case law has held that a detailed explanation of a complex medical procedure is more likely to bamboozle than inform,^108^ an unduly technical explanation of the computing processes underlying AI systems, robotics or related technologies would be counterproductive. A purposive interpretation of the consent requirement must focus on the need for the patient to understand enough about the risks of the process to make an informed decision about whether to proceed.^109^

The *National Health Act* 61 of 2003 sets out the principle that the “user”^110^ of health care services is to have “full knowledge”^111^ in that the health care provider must *inter alia* inform the “user” of “the range of diagnostic procedures and treatment options generally available”^112^ and the “benefits, risks, costs and consequences generally associated with each option”,^113^ as well as any implications, risks or obligations arising from the “user’s” exercise of the right to refuse treatment.^114^ Moreover the explanation must “where possible” be given in a language and in a manner that the user can understand.^115^ This qualification is a paradox. Informed consent simply cannot take place where the patient has not understood the explanation. South African law requires that the patient have “full knowledge” and there is a statutory,^116^ common law^117^ and ethical duty^118^ to obtain informed consent. How this requirement is to be met in practice requires careful consideration. Besides the obvious difficulties of explaining complex technologies in understandable terms, we must also explain what is presently unknown. Providing the patient with *full* knowledge may paradoxically require explaining that even the developers of the software and the treating doctors do not always fully understand the inner algorithmic workings of the AI.^119^ Further, we must put in place mechanisms to provide patients with additional information when it becomes available, and to obtain informed consent for sharing clinical data for research and development.^120^ Electronic patient consent and record management systems make this feasible.^121^

### 6.2 The primary health care practitioner bears legal responsibility

As illustrated above, the assumption underlying the existing legislation and ethical guidelines in health care in South Africa is that all instances of patient diagnosis and treatment are mediated through a human health care practitioner registered with the HPCSA in terms of the *Health Professions Act* 56 of 1974. In many instances this will continue to be the case and therefore, no matter how complex the AI system may be, “the last call”^122^ rests with the human health care practitioner.

At common law a health care practitioner’s liability when a treatment or diagnosis causes harm to a patient is based on the *Aquilian* action and involves applying a test for negligence based on an interrogation of what a reasonable medical professional ought to have done in the same situation.^123^

There is no reason to relax the ordinary standard of professional conduct because of the limitations of the technology or medium of communication used. A doctor could be found liable for harm on common law fault-based principles for failing to apply his or her own mind to the diagnosis or recommendations generated by the AI-software. The HPCSA guidelines state that professional discretion in relation to the course and scope of treatment “should not be limited by nonclinical considerations”^124^ such as the constraints of any technology. The consulting health care practitioner is also responsible for ensuring that the patient’s well-being comes first, and the patient’s rights to privacy, dignity, information about their condition and confidentiality are respected by servicing health care practitioners.^125^ They must ensure that adequate measures are in place to ensure the quality of service, as well as the confidentiality and security of the patient’s information, both in respect of their own employees as well as of non-health care personnel providing auxiliary or technical services,^126^ the optimal functioning of the technology,^127^ unauthorised access to patient information,^128^ and damage to or the loss or alteration of patient information.^129^

Thus, when a servicing health care practitioner is consulted the primary health care practitioner remains responsible. The primary health care practitioner must interpret and apply his or her own mind to results in advising a patient on treatment options, risk, and likely outcomes. By analogy, when AI systems are used the health care practitioner remains liable for errors and omissions in a diagnosis or treatment that were reasonably foreseeable^130^ or would not have been made by a reasonable practitioner in the same branch of the profession.^131^ Likewise the practitioner remains liable for a failure to obtain informed consent from the patient.^132^ To the extent that a greater degree of skill and care is required in the use of new and complex AI technologies, the practitioner would be expected to meet this higher standard,^133^ and could face civil or even criminal liability for the consequences of acting without the required knowledge and skill in the use of new technologies.^134^

There is, however, no guidance in case law on how to apply the principles of fault-based liability in a scenario where the outcome is primarily attributable to an unknown flaw or failing in the AI system that could not reasonably have been anticipated. One could theorise that if there is no causative fault on the part of the doctor,^135^ he or she would escape liability altogether, with the unfavourable outcome that the injured patient is left without recourse.^136^ Even if one turned to the legal doctrine of vicarious liability, there would be great difficulty in establishing, firstly, that the AI system “acted negligently” and, secondly, that the medical practitioner exerted a sufficient degree of control over the AI system to be held responsible.^137^ Moreover, one may well see an increase in the use of contractual exemption clauses to exclude all liability, save where the harm was intentionally caused,^138^ which all points to the need for clear legislative and policy guidelines to be developed in this area.

## 7 Opening the black box: an argument for strict liability

The principle of “explainability” requires that AI developers give clear, understandable explanations of how the algorithms function and present results to data protection and consumer protection authorities and the end user.^139^ This is the bedrock of consumer trust in new technologies, “even if the degree of [explicability] is relative to the complexity of the technologies”.^140^ Nevertheless, it is impossible in some cases even for the developer of the technology to explain how an algorithm arrived at a particular result,^141^ and this has given rise to the term the “black box algorithm”.^142^

### 7.1 Strict liability for operators of AI technology

When the machine makes a mistake that cannot be anticipated or explained, this raises difficulties about how to apply the common law of fault-based liability to the human health care practitioner. In simple terms, the doctor cannot be held liable on any standard of reasonableness. Moreover, the existing statutory and ethical framework does not impose any duty of care on the developers of AI applications in health care to prevent harm or obtain informed consent from the users of those technologies. At common law there is no general duty to prevent harm to others; and liability can be imputed for conduct only that is found to be wrongful when tested against the legal convictions of the community and the values embodied in the Constitution.^143^ In addition, causative fault in the form of negligence or intentional wrongdoing must be proved.

While there is a basis for imposing strict liability for high-risk activities under South African common law,^144^ legislation developed for the health care sector would be preferable in that it would provide a clear and certain framework to facilitate widespread adoption of and trust in such new technologies by health care practitioners and patients.

The latest EU legislative proposal on civil liability generally proposes joint and several fault-based liability on the operator(s) of AI systems.^145^ Health is classed as a “high risk” use case based on the sensitivity of health data and the potential for harm and the infringement of human rights, alongside consideration of the specific purpose or proposed use of the technology in any particular case, as well as the severity of possible harm.^146^ For this reason, strict liability (and mandatory insurance schemes) for health care practitioners are under consideration.^147^

### 7.2 Liability of developers and manufacturers of AI technologies

#### 7.2.1 Product liability

At common law, when a product fails liability is attributed either under the terms of the supply contract, using contractual warranties and service level agreements, or through the imposition of fault-based product liability for manufacturers and so-called expert retailers. This presented an “often insurmountable challenge”.^148^ For the non-lawyer, the term fault-based liability refers to the requirement that in addition to providing that the product was defective and caused harm, the claimant must prove that the supplier was negligent by failing to act in a reasonable manner and that the harm was caused by this negligence. Fault-based liability must therefore be distinguished from strict-liability, in terms of which a supplier is liable even if there was no fault.

One solution being considered in Europe is the application of the existing provisions of statutory product liability regimes, subject to appropriate amendments to incorporate digital goods and services within the ambit of the legislation.^149^

Product liability is governed in South Africa by the *Consumer Protection Act* 68 of 2008.^150^ Section 61 of the Act attempts to impose strict liability for product defects upon all parties in the supply chain, which would in theory include manufacturers, doctors, and hospitals. However, the Act provides for several defences that considerably vitiate its effectiveness.^151^

The Act also provides for a statutory warranty of quality and safety enforceable jointly and severally against “the producer or importer, the distributor and the retailer” but only for six months after purchase.^152^ Leaving aside the limited scope and duration of the warranty, the first problem is that the provision of goods and services to the State falls outside the ambit of the Act.^153^ There are also problems with the statute’s scope of application to private sector health care. Patients are unlikely to be parties to any transaction supplying AI software as a medical device (save in relation to mobile apps and wearable health monitors), although they may be able to claim protection under the Act as the term “consumer” is defined widely to include the end-user of the product or the recipient or beneficiary of the service,^154^ and would in those instances most likely seek to claim against the health care practitioner.^155^ When the health care practitioner uses AI technology in the course of performing a health care service or at any health care facility, the provisions of section 58(1) require that “any risk of an unusual character or nature” be disclosed, potentially widening the ambit of the informed consent obligations.^156^ The health care practitioner or facility that has purchased or used the AI technology will ordinarily be unable to rely on the Act for recourse against the developer. The Act’s protections apply to a consumer, and its provisions do not apply to a juristic person (which includes partnerships) with an annual turnover above R2 million.^157^ The application of the *Consumer Protection Act* 68 of 2008 to AI is thus an area requiring further research and possible reform.

### 7.2.2 Cross border enforcement difficulties

The first obvious problem with any proposal to impose liability on developers is that most AI applications will be developed outside South Africa. The solution in the Telemedicine guidelines is that

the practice of medicine takes place where the patient is located at the time the telemedicine technologies are used.^158^

This simple solution remains fit for purpose in relation to the liability of the health care practitioners treating the patient if it is extended to include all AI, robotics, and related technologies. However, for the purposes of establishing jurisdiction over the developer or deployer of such technology, or service-providers processing or storing the data on their behalf, it is inadequate. The elegant solution in article 3 of the EU Framework proposal could be considered as a model for a similar South African regulation:

This regulation applies to artificial intelligence, robotics and related technologies, where any part thereof is developed, deployed or used in the Union, regardless of whether the software, data or algorithms used or produced by such technologies are located outside of the Union or do not have a specific geographical location.

The provision overcomes the difficulties associated with the fact that technology components may be developed, manufactured, deployed, and operated by multiple parties in multiple jurisdictions. Pinning down the place where the cause of action arose and establishing personal jurisdiction over the responsible parties by the application of ordinary common law principles of jurisdiction may be cumbersome, if not impossible in some cases. While jurisdiction is commonly settled by agreement and recorded in the terms of the contact between the parties, this may also be an inadequate solution if it limits South Africans who have suffered harm to rights to action in a foreign court, where the cost and difficulty of enforcing their rights may render the rights nugatory.

### 7.2.3 Policy considerations

Competing policy considerations must be carefully weighed up, which in the field of health care include not only the protection of the individual but the broader policy goals of innovation and the widespread, cost-effective availability of new technologies.^159^ On the one hand, onerous strict liability regimes that leave health care practitioners with no recourse to claim an indemnity from the developers or manufacturers of AI products are unduly burdensome.^160^ Doctors and health facilities must rely on contractual service level agreements, software and hardware warranties and indemnity clauses to seek recourse against the supplier of AI products, or compulsory insurance schemes must be in operation which may in themselves be prohibitively costly. On the other hand, to impose direct liability on manufacturers and developers or to overregulate the field may stifle innovation, investment and SMME participation.^161^

## 8 The importance of a human rights-centred narrative in national policy

South Africa presently has no overarching national AI strategy, which contrasts poorly with the approach in countries such as Canada^162^ and China,^163^ that are moving forward swiftly with a 4IR policy agenda. The reports for the 4IR commission and the work of C4IR and ASSAf are moving in this direction. However, it is imperative that technical frameworks be developed in tandem with the guiding ethical principles and the review of the legal frameworks.

At their core, ethical AI principles seek to defend human autonomy, which is the very essence of the rights to dignity and privacy,^164^ against machine profiling and the practices it enables, which range from the somewhat innocuous (even helpful) functions of behaviourally targeted advertising and content suggestions to the subtle and insidious re-enforcement of hidden bias and discrimination. The cornerstone of a human rights-centred regulatory framework is the recognition that AI is made by people for people. It should therefore be designed “to serve people and not to replace or decide for them.”^165^

The regulation of AI in health care must therefore take due cognisance of the constitutional rights of dignity^166^ and privacy,^167^ alongside equality,^168^ life,^169^ bodily and psychological integrity,^170^ access to health care services, including reproductive health care,^171^ and access to information,^172^ as well as the rights in the Patient’s Rights Charter,^173^ including the right to the confidentiality of one’s information required by the *National Health Act* 61 of 2003. There is a strong alignment between the international normative framework of principles for ethical AI development and the rights in the Bill of Rights under the Constitution of South Africa.

There is a robust body of constitutional case law recognising that there is a “strong privacy interest” in maintaining the confidentiality of health information,^174^ and that

[t]he more intimate that information, the more important it is in fostering privacy, dignity and autonomy that an individual makes the primary decision whether to release the information. That decision should not be made by others.^175^

However, the conceptualisation of privacy purely in terms of the right to decide whether to disclose data at all, for example, must make way to permit the free flow of data for research and innovation but still respect the individual’s human rights. In doing so the central challenge to the ethical development of AI is to ensure that we do not reduce the human being to an object “to be sifted, sorted, scored, herded, conditioned or manipulated.”^176^ A human rights-centred narrative in any AI strategy is thus essential.

South Africa’s digital health strategy places a “person-centred focus” as the first of five key principles underpinning the strategy^177^ and highlights the need for digital health solutions to respect “patient privacy”.^178^ The report by the Presidential Fourth Industrial Revolution Commission^179^ recognises that AI could herald great advances in health care but that “the data ecosystem also brings about the critical need for policy and legislation relating to the use of data, including ethics and security.”^180^ Referring to the “central productive force of data”^181^ in the 4IR, the report recognises

perhaps more importantly, that fundamental human rights are now intertwined with the protection of data.^182^

The danger I point out is that trite references in passing to “patient privacy” are insufficient, and a clear commitment to and detailed treatment of human rights issues such as that contained in the EU “trustworthy AI” approach^183^ is required.

## 9 Conclusion

South Africa has neither an overarching AI strategy nor any specific laws governing AI. Although there may be some temptation to adopt a “wait and see” approach,^184^ early and proactive engagement in the regulatory endeavour is important to ensure that laws are not Western “imports” but are fashioned to be appropriate to the South African context.^185^

The development of a national policy framework of guiding ethical principles would in no way undermine the existing legislation and ethical guidelines governing health care practitioners, which must be read alongside AI guidelines, and implemented to their full effect.^186^

This article has examined three key areas for legal reform in relation to AI in health care. The first is that the regulatory framework for the oversight of software as a medical device needs to be updated to develop frameworks for adequately regulating the use of such new technologies. In this regard the WHO framework^187^ provides a solid starting point for the planning of clinical and research studies and the reform of South Africa’s regulatory system to accommodate AI software as a medical device.

Secondly, the present HPCSA guidelines for health care practitioners in South Africa adopt an unduly restrictive approach centred in the outmoded semantics of telemedicine. This may discourage technological innovation that could improve access to health care for all, and as such the guidelines are inconsistent with the national digital health strategy. As a first step, such guidelines should be amended to expressly permit the use of AI and to provide additional guidance on informed consent in such contexts.

Thirdly, the common law principles of fault-based liability for medical negligence could prove inadequate to providing patients and users of new technologies with redress for harm. Consideration should be given to developing a statutory scheme for strict liability, together with mandatory insurance, and the appropriate reform of product liability pertaining to technology developers and manufacturers. It is suggested that the EU model should be considered as a starting point for developing an AI Act for South Africa.

These legal reforms should not be undertaken without also developing a coherent, human rights-centred policy framework for the ethical use of AI, robotics, and related technologies in health care in South Africa.

## Footnotes

1 WMA 2017 https://www.wma.net/policies-post/wma-declaration-of-geneva/.

2 Report of the Presidential Commission on the Fourth Industrial Revolution (4IR) (in GN 591 in GG 43834 of 23 October 2020) 26, after a survey of 4IR strategy in 13 nations. AI is a focus area of the Centre for 4IR (C4IR) operated by the Council for Scientific and Industrial Research as an affiliate of the Centre for the Fourth Industrial Revolution Network (C4IR Network) launched by the World Economic Forum in March 2017.

3 ASSAf 2018 http://dx.doi.org/10.17159/assaf.2018/0033.

4 Jobin, Ienca and Vayena 2019 *Nature Machine Intelligence* 389.

5 DuBois, Chibnall and Gibbs 2016 *Sci Eng Ethics* 966.

6 Townsend 2020 *TSAR* 759.

7 Ameer-Mia, Pienaar and Kekana “South Africa” 248–249; Singh 2020 https://policyaction.org.za/sites/default/files/PAN_TopicalGuide_AIData6_Health_Elec.pdf.

8 DuBois, Chibnall and Gibbs 2016 *Sci Eng Ethics* 967.

9 Ormond 2020 *The Thinker* 5. As to the challenges, see Oxford Insights 2019 https://africa.ai4d.ai/wp-content/uploads/2019/05/ai-gov-readiness-report_v08.pdf; UNESCO 2019 https://unesdoc.unesco.org/ark:/48223/pf0000374014; UNESCO 2021 https://unesdoc.unesco.org/ark:/48223/pf0000375322.

10 Access Partnership and University of Pretoria 2018 https://www.up.ac.za/media/shared/7/ZP_Files/ai-for-africa.zp165664.pdf.

11 Wiegand *et al*. date unknown https://www.itu.int/en/ITU-T/focusgroups/ai4h/Documents/FG-AI4H_Whitepaper.pdf 8.

12 Microsoft 2019 https://info.microsoft.com/rs/157-GQE-382/images/MicrosoftSouthAfricanreportSRGCM1070.pdf.

13 UNAIDS date unknown https://www.unaids.org/en/resources/909090.

14 BroadReach Healthcare 2019 https://www.broadreachcorporation.com/south-africa-leading-the-way-in-the-fight-against-hiv-and-aids/. But see Cleary 2020 https://www.spotlightnsp.co.za/2020/03/18/special-investigation-claims-of-90-90-90-success-in-kzn-districts-were-premature/ – reports that the targets have been met might be premature in the face of evidence on the ground from social workers.

15 Promoting the adoption of information and communication technologies (ICTs) by small, micro and medium enterprises (SMMEs) is a key national policy objective: National Integrated ICT Policy White Paper in GN 1212 in GG 40325 of 3 October 2016; National E-Strategy in GN 343 in GG 40772 of 7 April 2017; National E-Government Strategy and Roadmap in GN 341–342 in GG 40772 of 7 April 2017.

16 For a review of promising studies see Wiegand *et al*. date unknown https://www.itu.int/en/ITU-T/focusgroups/ai4h/Documents/FG-AI4H_Whitepaper.pdf 3. For further examples of how AI was used in healthcare in response to the COVID-19 pandemic, see Mahomed 2020 *SAMJ* 2; ITU-T 2020 https://www.itu.int/en/ITU-T/focusgroups/ai4h/Documents/FGAI4H-DT4ER-O-001.pdf.

17 Walch 2020 https://www.forbes.com/sites/cognitiveworld/2020/02/20/ai-laws-are-coming/.

18 Article 4(a) of the *European Parliament Resolution of 20 October 2020 with Recommendations to the Commission on a Framework of Ethical Aspects of Artificial Intelligence, Robotics and Related Technologies* (European Parliament 2020 https://www.europarl.europa.eu/doceo/document/TA-9-2020-0275_EN.html) (hereafter EU Framework Resolution). Also see European Commission 2018 https://eur-lex.europa.eu/legal-content/EN/TXT/?uri=COM%3A2018%3A237%3AFIN 1.

19 OECD 2019 https://legalinstruments.oecd.org/en/instruments/OECD-LEGAL-0449 resolution I.

20 The term “big data” refers to data that has the three characteristics of a massive volume, the velocity of processing and the variety of data types processed. Townsend and Thaldar 2019 *SAJHR* 331; Donnelly *Privacy by (re)Design* 78–79.

21 Mahomed 2018 *SAJBL* 94.

22 Dourish 2016 *Big Data & Society* 3–6 explains the functioning of algorithms and their relation to source code, the distributed architecture of networked computing systems, and the constraints of specific instantiations of the abstract algorithm into a particular setting.

23 See the synopsis of Schönberger 2019 *Int J Law Inf Technol* 174–175.

24 Morley et al. 2019 https://ssrn.com/abstract=3486518 2.

25 For a classification of different machine-learning (ML) types, see Flach *Machine Learning*.

26 Jiang et al. 2017 *Stroke and Vascular Neurology* 231.

27 Townsend 2020 *TSAR* 749.

28 UNESCO 2017 https://unesdoc.unesco.org/ark:/48223/pf0000253952 4, 17.

29 CAHAI 2020 https://rm.coe.int/prems-107320-gbr-2018-compli-cahai-couv-texte-a4-bat-web/1680a0c17a.

30 Most notably human dignity and privacy, and the preservation of human autonomy that is encapsulated by these rights.

31 Jobin, Ienca and Vayena 2019 *Nature Machine Intelligence* 394–396; Hagendorff 2020 *Minds and Machines* 103; Fjeld et al. 2019 https://papers.ssrn.com/sol3/papers.cfm?abstract_id=3518482; Zeng, Lu and Huangfu 2018 https://arxiv.org/ftp/arxiv/papers/1812/1812.04814.pdf.

32 OECD 2019 https://legalinstruments.oecd.org/en/instruments/OECD-LEGAL-0449.

33 UNESCO 2021 https://unesdoc.unesco.org/ark:/48223/pf0000380455.

34 G20 2019 https://www.mofa.go.jp/files/000486596.pdf.

35 European Commission 2019 https://data.europa.eu/doi/10.2759/177365.

36 UNESCO 2021 https://unesdoc.unesco.org/ark:/48223/pf0000380455.

37 IEEE 2019 https://standards.ieee.org/industry-connections/ec/autonomous-systems.html.

38 Microsoft date unknown https://www.microsoft.com/en-us/ai/responsible-ai?activetab=pivot1%3aprimaryr6.

39 Google AI date unknown https://ai.google/principles/.

40 DeepMind date unknown https://deepmind.com/applied/deepmind-ethics-society/principles/.

41 Hagendorff 2020 *Minds and Machines* 108–109; Jobin, Ienca and Vayena 2019 *Nature Machine Intelligence* 389.

42 *Consumer Protection Act* 68 of 2008 (CPA).

43 *Protection of Personal Information Act* 4 of 2013.

44 *Promotion of Access to Information Act* 2 of 2000.

45 *Electronic Communications and Transactions Act* 25 of 2002.

46 DoH *National Digital Health Strategy* 9.

47 DoH *National e-Health Strategy* 15.

48 WHO *Global Strategy on Digital Health* 5. Digital health is used to refer to “the field of knowledge and practice associated with the development and use of digital technologies to improve health”.

49 WHO *Global Strategy on Digital Health* 5. eHealth is used to refer to the “use of information and communications technologies in support of health and health-related fields, including health care services, health surveillance, health literature, and health education, knowledge and research.”

50 WHO *Global Strategy on Digital Health* 6.

51 AU 2019 https://au.int/en/documents/20200518/digital-transformation-strategy-africa-2020-2030.

52 Research ICT Africa 2021 https://researchictafrica.net/2021/02/15/ria-provides-technical-assistance-for-development-of-aus-digital-health-strategy/.

53 DoH *National Digital Health Strategy* 9.

54 Section 1 of *National Health Act* 61 of 2003 (NHA).

55 As defined in s 1 of the *Medicines and Related Substances Act* 101 of 1965.

56 Sections 23(1)(a)(v) and 27(1)(a)(v) of the NHA.

57 Section 36 of the NHA.

58 Section 90(1)(r) of the NHA.

59 Sections 47(1) and (2) of the NHA.

60 Section 47(3) of the NHA.

61 Pillay 2019 https://mg.co.za/article/2019-11-22-00-the-future-of-health-in-south-africa/. Digital health records are used by fewer than 40% of South African health care practitioners. As to the challenges in implementing health technology policy also see Mueller 2020 *Int J Technol Assess Health Care*.

62 The amendments to the definition of “medical device” by s 1(h) of the *Medicines and Related Substances Amendment Act* 14 of 2015 are not material to this discussion. They extend the definition to include devices for use on animals and changed terminology referring to reagents for *in vitro* use.

63 Townsend 2020 *TSAR* 751.

64 Lang 2017 *JMIR Biomed Eng* 2.

65 Townsend 2020 *TSAR* 751.

66 Luxton 2020 *Bull World Health Organ* 286.

67 Section 2B(1)(a) of the *Medicines and Related Substances Act* 101 of 1965. The South African Health Products Regulatory Authority’s (SAHPRA) functions also relate to medicines and *in vitro* diagnostics but those are not considered in this article.

68 Section 13(2) of the *Medicines and Related Substances Act* 101 of 1965.

69 Section 13(1) of the *Medicines and Related Substances Act* 101 of 1965.

70 FDA 2021 https://www.fda.gov/medical-devices/software-medical-device-samd/artificial-intelligence-and-machine-learning-software-medical-device 2.

71 FDA 2021 https://www.fda.gov/medical-devices/software-medical-device-samd/artificial-intelligence-and-machine-learning-software-medical-device 2.

72 European Parliament 2017 https://eur-lex.europa.eu/legal-content/EN/TXT/? uri=CELEX%3A32017R0745 (hereafter Regulation (EU) 2017/745).

73 Lang 2017 *JMIR Biomed Eng*. Regulation (EU) 2017/745 recital 19, which excludes “general software” and “software intended for life-style and wellbeing purposes” from the scope of the regulation.

74 Regulation (EU) 2017/745 annex VIII rule 11.

75 European Commission 2021 https://eur-lex.europa.eu/legal-content/EN/TXT/?uri=CELEX%3A52021PC0206.

76 ITU/WHO date unknown https://www.itu.int/en/ITU-T/focusgroups/ai4h/Pages/default.aspx. In addition, the International Medical Device Regulators Forum (IMDRF) has established an AI working group, but South Africa’s medical regulator is neither a member nor an official observer.

77 WHO *Generating Evidence for Artificial Intelligence-based Medical Devices*.

78 WHO *Generating Evidence for Artificial Intelligence-based Medical Devices* 33.

79 Smit and Mwale 2019 *Without Prejudice*.

80 EU Framework Resolution para 20.

81 EU Framework Resolution para 20.

82 EU Framework Resolution paras 20 and 23.

83 EU Framework Resolution para 123.

84 EU Framework Resolution paras 125, 135–136.

85 EU Framework Resolution para 21.

86 HPCSA date unknown Booklet 10 https://www.hpcsa.co.za/Uploads/Professional_Practice/Ethics_Booklet.pdf 178 (hereafter HPCSA *Telemedicine*) para 3.1.

87 HPCSA *Telemedicine* para 3.1.

88 HPCSA *Telemedicine* para 1.3.

89 HPCSA *Telemedicine* paras 4.1.2–4.1.3.

90 HPCSA *Telemedicine* para 4.4.1. Also see Barit 2019 *SAMJ* 150; Mahomed 2018 *SAJBL* 95.

91 HPCSA *Telemedicine* para 4.4.2.

92 HPCSA *Telemedicine* para 4.4.3.

93 HPCSA 2020 https://www.hpcsa.co.za/Uploads/Events/Announcements/APPLICATION_OF_TELEMEDICINE_GUIDELINES.pdf clause (a) substitutes the term “telemedicine” with “telehealth” which “includes amongst others, Telemedicine, Telepsychology, Telepsychiatry, Telerehabilitation, etc., and involves remote consultation with patients using telephonic or virtual platforms of consultation”.

94 HPCSA 2020 https://www.saheart.org/cms/content/104-notice-to-amend-telemedicine-guidelines-during-covid-19-%E2%80%93-dated-3-april-2020-%7C-hpcsa-e-bulletin clause (b).

95 Sections 41–42 of the *Health Professions Act* 56 of 1974.

96 See e.g., *Jansen van Vuuren v Kruger* 1993 4 SA 842 (A) 850E-F, in relation to the duty of confidentiality. It does not follow from the dicta that every ethical duty will amount to an actionable delict under common law, but doctors also face professional sanction by the HPCSA.

97 DOH *National Digital Health Strategy* 18.

98 GN 591 in GG 43834 of 23 October 2020 30.

99 GN 591 in GG 43834 of 23 October 2020 63.

100 See e.g. Jiang *et al.* 2017 *Stroke and Vascular Neurology* 241 discussing the pioneering work in the field of oncology diagnosis of the IBM Watson system.

101 In South African law both natural and juristic persons can be the subject of legal rights and duties, including the “human” rights and corresponding duties created in the Bill of Rights. The *Constitution of the Republic of South Africa*, 1996 (the Constitution) s 8(2) provides: “A provision of the Bill of Rights binds a natural or a juristic person if, and to the extent that, it is applicable, taking into account the nature of the right and the nature of any duty imposed by the right.”

102 The reference to artificial (legal) persons in *Financial Mail (Pty) Ltd v Sage Holdings Ltd* 1993 2 SA 451 (AD) para 25 applied the right of privacy to a company.

103 Although the origins of the admiralty action *in rem* are lost in the mists of time, the *Admiralty Jurisdiction Regulation Act* 105 of 1983 permits the arrest of a ship which is cited as the defendant in proceedings and any judgment given on the claim. *Transnet Ltd v The Owner of the Alina II* 2011 6 SA 206 (SCA) para 29–30.

104 These are set out in ss 7(1)(a)-(c) of the *National Health Act* 61 of 2003, namely where the user is “unable to give informed consent”, authorisation by law or court order, a “serious risk to public health” or (where the patient has not refused the service) “death or irreversible damage to his or her health”.

105 HPCSA *Telemedicine* paras 4.4.3, 4.5.3 and 4.6. See further on the protection of information HPCSA date unknown *Booklet 5* https://www.hpcsa.co.za/Uploads/Professional_Practice/Ethics_Booklet.pdf.

106 *Protection of Personal Information Act* 4 of 2013 ss 17, 18 and 71; European Parliament 2016 https://eur-lex.europa.eu/eli/reg/2016/679/oj (EU GDPR) ss 12 and 22.

107 Morley et al. 2020 *Sci Eng Ethics* 2155.

108 Schönberger 2019 *Int J Law Inf Technol* 188.

109 *Castell v De Greef* 1994 4 SA 408 (C) 425H-I/J, in which it is held that informed consent requires knowledge and appreciation of the nature and extent of the harm or risk.

110 The patient, as the “user” of a health care service as defined in s 1 of the NHA, is also the “data subject”, being the person to whom the personal health information relates, under the *Protection of Personal Information Act* 4 of 2013. The latter Act also imposes additional stipulations for the processing of health data and other “special” personal information.

111 NHA s 6.

112 NHA s 6(b).

113 NHA s 6(c).

114 NHA s 6(d).

115 NHA s 6(2).

116 NHA s 7.

117 *Castell v De Greef* 1994 4 SA 408 (C).

118 HPCSA date unknown *Booklet 1* https://www.hpcsa.co.za/Uploads/Professional_Practice/Ethics_Booklet.pdf item 5.3; HPCSA date unknown *Booklet 4* https://www.hpcsa.co.za/Uploads/Professional_Practice/Ethics_Booklet.pdf.

119 Gerke, Minssen and Cohen “Ethical and Legal Challenges” 310 outlines three aspects on which guidance is needed: when it must be disclosed that AI is being used, to what extent the clinician has a responsibility to explain the complexities of the AI to the patient, and if the limits of the doctor’s own understanding of the AI must be disclosed. These questions also need to be addressed in healthcare settings that are not mediated through a traditional doctor-patient relationship, such as the use of health apps and chatbots. See McPake 2020 https://medium.com/frontier-technologies-hub/pilot-story-will-access-to-sex-positive-and-reproductive-health-information-through-a-chatbot-d41738947d0c.

120 The requirement to obtain informed consent for the collection of any personal data (even if it will be shared only in anonymised form) must be adhered to in clinical and research settings. Such matters are regulated in South Africa by the *Protection of Personal Information Act* 4 of 2013. Also see HPCSA *Telemedicine* para 4.6.

121 In a telemedicine setting consent must be in writing. HPCSA *Telemedicine* paras 4.6.2 and 4.6.5. An electronic data message and electronic signature are valid in terms of ss 12 and 13 respectively of the *Electronic Communications and Transactions Act* 25 of 2002.

122 Schönberger 2019 *Int J Law Inf Technol* 191.

123 McQuoid-Mason 2010 *SA Heart*.

124 HPCSA *Telemedicine* para 4.2.5. The situation where reliance was reasonably placed on the technology and harm results from some failure that could not be reasonably anticipated and avoided is considered in section 7.1.

125 HPCSA *Telemedicine* para 4.3.2(a).

126 HPCSA *Telemedicine* paras 4.7.5, 4.7.6, 4.9.1 and 4.9.4.

127 HPCSA *Telemedicine* para 4.9.5 (a)-(b).

128 HPCSA *Telemedicine* para 4.9.6.

129 HPCSA *Telemedicine* para 4.9.7.

130 *Richter v Estate Hamman* 1976 3 SA 226 (C).

131 *Mitchell v Dixon* 1914 AD 519.

132 *Castell v De Greef* 1993 3 SA 501 (C); *Dube v Administrator Transvaal* 1963 4 260 (T).

133 *Van Wyk v Lewis* 1924 AD 438 lays down the general principle that a greater degree of skill and care is required to perform complex procedures. Of course, in future, as AI technologies become commonplace, it may come to pass that it is regarded as negligent to diagnose or treat a patient without making use of AI.

134 *S v Mkwetshana* 1965 2 SA 493 (N) concerned a junior doctor charged with culpable homicide for the death of a patient caused by the administration of the incorrect dosage of a drug. By analogy, administering any medical treatment that requires an expert skill that the doctor is lacking would lead to liability.

135 Shortcomings in conduct do not give rise to legal liability in the absence of proof of causative fault, no matter how great the suffering of the blameless patient may be: Broude v McIntosh 1998 3 SA 60 (SCA) 75B; *Michael v Linksfield Park Clinic (Pty) Ltd* 2001 3 SA 1188 (SCA).

136 The principle that the loss lies where it falls applicable at common law holds that a person must bear any injury suffered unless there was both a duty on another person to prevent the injury, and failure by that person to act reasonably in the discharge of the duty of care caused the injury.

137 By analogy in the operating theatre a surgeon may be held vicariously liable for the negligence of his or her theatre nurse, but not for the negligence of the anaesthetist, unless the doctor could have acted to prevent the harm. *S v Kramer* 1987 1 SA 887 (W).

138 As to the validity of such clauses, see *Afrox Healthcare Bpk v Strydom* 2002 6 SA 21 (SCA). The judgment was, and remains, controversial. This only strengthens arguments for *sui generis* AI legislation to address the necessary balance between public benefit from technological innovation and patient safety and privacy concerns.

139 EU Framework Resolution paras 17–18.

140 EU Framework Resolution para 23.

141 EU Framework Resolution para 23.

142 The term is a reference to the fact that the inputs (data) and outputs (diagnosis) of the machine are known, but the inner logic by which it reached that conclusion is inscrutable. Watson et al. 2019 *BMJ* 365.

143 *Oppelt v Head: Health, Department of Health Provincial Administration: Western Cape* 2016 1 SA 325 (CC) para 51.

144 Neethling and Potgieter *Law of Delict* 380; Loubser *et al. Law of Delict* 458.

145 European Parliament 2020 https://www.europarl.europa.eu/doceo/document/TA-9-2020-0276_EN.html (hereafter EU CL).

146 Annex to EU Framework Resolution and EU CL.

147 EU CL paras 24–26.

148 Gowar 2011 *Obiter* 536.

149 EU CL para 9 proposes that this be accommodated under reforms of the product liability directive: Council of the European Committees 1985 https://eur-lex.europa.eu/legal-content/EN/TXT/PDF/?uri=CELEX:31985L0374&from=EN 29–33. For further discussion of the EU position see Cabral 2020 *MJ*. Also see Alheit 2001 *CILSA* 199 *et seq*. for a discussion of when software is “defective”.

150 Product liability, which is concerned with harm resulting from defects in goods such as the AI-software or medical robot, must in turn be distinguished from liability for harm arising from services. The CPA does also apply to services, and although it does not impose strict liability for harm arising from the provision of a service per se, s 54(1)(c) of the Act provides that, when those services involve the use or supply of goods, the goods must be free of defects.

151 CPA s 61(4). Notably s 61(4)(c) muddies the water by providing that it is a defence if the person could not reasonably have known of the defect. It is also open to argue that when AI software is approved by SAHPRA (as it must be), then s 61(4)(a) provides a complete defence to damages claims on the grounds that the product defect is “wholly attributable to compliance with any public regulation”, and likewise s 61(4)(b)(ii), which applies when the product was operated in accordance with the supplier’s instructions.

152 CPA s 56.

153 CPA s 5(1)(a).

154 The CPA definition of “consumer” para (c).

155 Nöthling-Slabbert and Pepper 2011 *SAMJ* 801.

156 Nöthling-Slabbert et al 2011 *CILSA*.

157 CPA s 5(1)(b), based on the current threshold value of an annual turnover of R2 million.

158 HPCSA *Telemedicine* para 4.2.2.

159 Foote “Product Liability and Medical Device Regulation” 73–92.

160 EU CL rec 13.

161 EU CL rec 3 records that a balance must be struck between protecting the public and not creating stifling “red tape” that might discourage investment and innovation. At the same time the EU CL records in the preamble para (K) that “legal certainty is also an essential precondition for dynamic development and innovation of AI-based technology.”

162 CIFAR date unknown https://cifar.ca/ai/.

163 Roberts et al. 2021 *AI & Society*.

164 Khumalo v Holomisa 2002 5 SA 401 (CC) para 27 affirmed that “The value of human dignity in our Constitution is not only concerned with an individual’s sense of self-worth, but constitutes an affirmation of the worth of human beings in our society. … The right to privacy, entrenched in section 14 of the Constitution, recognises that human beings have a right to a sphere of intimacy and autonomy that should be protected from invasion. This right serves to foster human dignity. No sharp lines then can be drawn between reputation, *dignitas* and privacy in giving effect to the value of human dignity in our Constitution.” Also see *National Coalition for Gay and Lesbian Equality v Minister of Justice* 1999 1 SA 6 (CC) para 30.

165 See EU Framework Resolution para 2. Also see paras 10–11 identifying human well-being, individual freedom and international peace and security as the guiding objectives for the development and deployment of AI, and the need for mechanisms to ensure human agency, oversight and resumption of control.

166 Section 10 of the Constitution.

167 Section 14 of the Constitution.

168 Section 9 of the Constitution.

169 Section 11 of the Constitution.

170 Section 12(2) of the Constitution protects one’s security in and control over one’s body and the need for informed consent for any decisions made about what happens to it.

171 Section 27(1)(a) of the Constitution.

172 Section 32 of the Constitution.

173 HPCSA date unknown *Booklet 3* https://www.hpcsa.co.za/Uploads/Professional_Practice/Ethics_Booklet.pdf.

174 *NM v Smith* 2007 5 SA 250 (CC) para 41, cited with approval in *Tshabalala-Msimang v Makhanya* 2008 6 SA 102 (W) 114A.

175 *NM v Smith* 2007 5 SA 250 (CC) para 132.

176 European Commission 2019 https://data.europa.eu/doi/10.2759/177365 10.

177 DoH *National Digital Health Strategy* 18 defines the approach as one in which “all individuals and their families are involved in and able to influence the health care required, thus leading to interventions that better meet their unique needs.”

178 DoH *National Digital Health Strategy* 14, referring to WHO *Recommendations on Digital Interventions*.

179 GN 591 in GG 43834 of 23 October 2020.

180 GN 591 in GG 43834 of 23 October 2020 209.

181 GN 591 in GG 43834 of 23 October 2020 209.

182 GN 591 in GG 43834 of 23 October 2020 209.

183 European Commission 2019 https://data.europa.eu/doi/10.2759/177365.

184 Marwala 2020 https://mg0.co.za/article/2020-04-03-review-amend-or-create-policy-and-legislation-enabling-the-4ir/.

185 Vawda and Shozi 2020 https://ssrn.com/abstract=3559478.

186 A point emphasised in EU Framework Resolution para 146, and recital 5.

187 WHO *Generating Evidence for Artificial Intelligence-based Medical Devices*.

## List of Abbreviations

4IR: Fourth Industrial Revolution
AI: artificial intelligence
ASSAf: Academy of Science of South Africa
AU: African Union
BMJ: British Medical Journal
Bull: World Health Organ Bulletin of the World Health Organization
C4IR: Centre for the 4IR
CAHAI: Council of Europe’s Ad Hoc Committee on Artificial Intelligence
CILSA: Comparative and International Law Journal of Southern Africa
CPA: Consumer Protection Act 68 of 2008
DOH: Department of Health, South Africa
EU: European Union
FDA: United States Food and Drug Administration
GDPR: General Data Protection Regulation
HPCSA: Health Professions Council of South Africa
ICT: information and communications technology
IEEE: Institute of Electrical and Electronics Engineers
Int J Law Inf Technol: International Journal of Law and Information Technology
Int J Technol Assess Health Care: International Journal of Technology Assessment in Health Car
ITU-T: International Telecommunication Union
JMIR Biomed Eng: JMIR Biomedical Engineering
MJ: Maastricht Journal of European and Comparative Law
ML: machine-learning
NHA: National Health Act 61 of 2003
OECD: Organisation for Economic Co-operation and Development
SAHPRA: South African Health Products Regulatory Authority
SAJBL: South African Journal of Bioethics and Law
SAJHR: South African Journal on Human Rights
SAMJ: South African Medical Journal
Sci Eng Ethics: Science and Engineering Ethics Journal
SMME: small medium and micro-sized enterprise
TSAR: Tydskrif vir die Suid-Afrikaanse Reg
UNESCO: United Nations Educational, Scientific and Cultural Organization
WHO: World Health Organization
WMA: World Medical Association
